# Gaps in Palliative Care Education among Neonatology Fellowship Trainees

**DOI:** 10.1089/pmr.2021.0011

**Published:** 2021-07-27

**Authors:** Catherine Lydia Wraight, Jens C. Eickhoff, Ryan M. McAdams

**Affiliations:** ^1^ Division of Neonatology, Department of Pediatrics, and University of Wisconsin, Madison, Wisconsin, USA.; ^2^ Department of Biostatistics and Medical Informatics, University of Wisconsin, Madison, Wisconsin, USA.

**Keywords:** curriculum, medical education, NICU, residency, training program

## Abstract

***Background:*** To provide proper care for infants at risk for death, neonatologists need expertise in many areas of palliative care. Although neonatology training programs have implemented a wide variety of palliative care educational programs, the impact of these programs on trainees' skills and effective communication regarding end-of-life issues remains unclear.

***Objective:*** To determine whether neonatology fellowship programs are providing formal palliative care education and assess whether this education is effective at increasing fellows' self-reported comfort with these important skills.

***Methods:*** An anonymous survey was sent to program directors (PDs) and fellows of ACGME accredited neonatology fellowship programs in the United States. Using a 5-point Likert scale, participants were asked about the palliative care education they received, and their comfort level with several key aspects of palliative care.

***Results:*** Twenty-four (26%) PDs and 66 (33%) fellows completed the survey. Fourteen PDs (58%) reported including palliative care education in their formal fellowship curriculum, whereas only 20 (30%) responding fellows reported receiving palliative care education. Of the responding fellows, most (80%) reported being uncomfortable or only somewhat comfortable with all assessed areas of palliative care. Fellows who received formal education were more comfortable than those without it in leading goals of care conversations (*p* = 0.001), breaking bad news (*p* = 0.048), discussing change in code status (*p* = 0.029), and grief and bereavement (*p* = 0.031).

***Conclusions:*** Most fellows report being uncomfortable or only somewhat comfortable with essential areas of palliative care. Formal palliative care education improves fellows' self-reported comfort with important aspects of end-of-life care. To promote a well-rounded neonatology fellowship curriculum, inclusion of formal palliative care education is recommended.

## Introduction

Neonatologists, and neonatology fellows, are at the front line of providing care when infants die. Approximately one-third of pediatric, and two-thirds of infant deaths in the United States occur in the neonatal period,^1,2^ and the majority of these deaths occur in the hospital.^3^ The importance of supporting children and families through critical, complex illness, and at the end of life has been recognized by the American Academy of Pediatrics,^4^ as well as the American Board of Pediatrics (ABP).^5^ However, although the value of palliative care has been increasingly accepted, in many fields of medicine, training remains inconsistent and potentially inadequate.^6,7^ Studies of pediatric residency programs have found that trainees feel unprepared to provide quality end-of-life care and desire further education on these topics^8^; however, few studies have looked specifically at pediatric fellows.

Although neonatologists report caring for dying infants frequently, they often have received little or no training in end-of-life care,^9–11^ or how to support a family through child loss. Dealing with dying critically ill babies is intense; this is an incredibly important part of neonatology and has a major impact on the dying baby, their families, and the health care providers in the neonatal intensive care unit (NICU). Fellowship programs have increasingly incorporated aspects of palliative care into their training curricula.^9,12^ However, there is currently no recommended way to teach neonatal fellows the appropriate skills needed to care for dying infants and their families. Further, the educational methods and content vary greatly between programs.

The crucial emotional and psychological aspects surrounding the care of a dying baby require a thoughtful, individualized, and delicate approach. Given our experiences as neonatologists, we suspected that not all neonatology fellowship programs provide formal palliative care education. This prompted us to conduct our survey to determine the extent to which neonatology fellowship training programs are providing formal palliative care education to their trainees, as well as to assess whether this education is effective in increasing fellows' self-reported comfort with these important skills.

## Materials and Methods

**FIG. 1.**
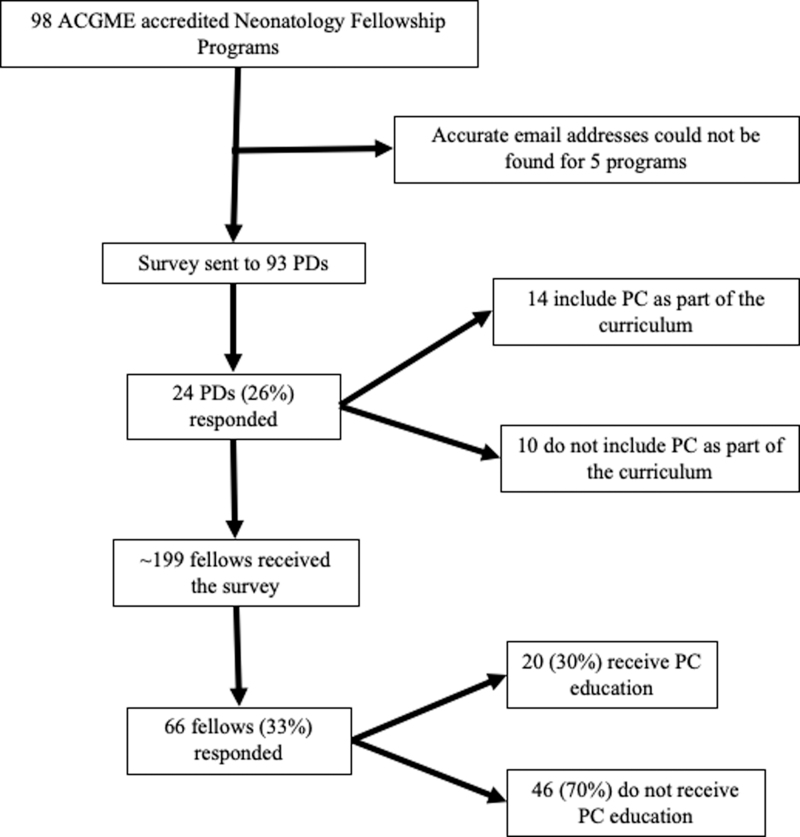
Identification and distribution of survey respondents. PD, program director; PC, palliative care.

An electronic survey tool (Supplementary Appendix SA1) was developed in Research Electronic Data Capture (REDCap)^13,14^ by the researchers, and it was vetted by a panel of neonatologists and neonatology fellows at the University of Wisconsin-Madison. A new tool was designed, because no current tool was available that covered all of the areas of interest specific to pediatric trainees and neonatology. Neonatology fellowship training programs were identified through the FREIDA Residency Program Search website. Program directors (PDs) were confirmed on the ABP website and individual program websites. Ninety-eight ACGME accredited neonatology fellowship programs were identified. Programs for which a current e-mail address for the PD was not available were excluded (Fig. 1). After receiving an exemption from our internal review board, an anonymous survey link was distributed via e-mail to the PDs of 93 neonatology fellowship programs. The survey was sent a total of three times, at ∼2-week intervals, and accompanied by a cover letter explaining the project. The PDs were asked to forward the survey link to their fellows and were asked how many fellows they had in their program.

All respondents were asked several demographic questions, including their role in the NICU. The PDs were asked about what types of palliative care education were provided to their fellows. Fellows were asked about the education they received and, using a 5-point Likert scale, about their comfort level with several key aspects of providing palliative care. The PDs and fellows who reported either providing or receiving palliative education were asked about the educational formats used. Commonly used formats in palliative care education, such as lectures or seminars, clinical time with a palliative care provider, workshops, simulation or role-play, and online modules, videos, or other self-study, were provided as options. On-the-job training was not included due to the inconsistent and unpredictable nature of direct patient care.

Demographic variables were summarized in terms of frequencies and percentages. Fellow self-reported comfort level 5-point Likert scale responses were categorized into “Very Comfortable” versus all others. This comparison was chosen, because it was the most likely response to have resulted from specific education in the area of palliative care, rather than the much less specific response of “somewhat comfortable,” which has a much wider interpretation. Response comparisons between fellows with formal palliative care training and fellows without formal palliative care training were conducted by using a chi-square or Fisher's exact test. All reported *p*-values are two-sided, and *p* < 0.05 was used to define statistical significance. Statistical analysis was conducted by using SAS software (SAS Instituted, Cary, NC) version 9.4.

## Results

Surveys were completed by 26% (24/93) of PDs. All responding PDs reported sending the survey to their fellows; based on this, it was estimated that ∼199 fellows were sent the survey, of whom 66 (33%) completed it (Fig. 1). The majority of respondents were from the Midwest and New England areas; however, a variety of regions were represented (Supplementary Appendix SA2). The responses of PDs could not be linked to the responses of their fellows. Due to this, and due to the small sample size, statistical comparison was not possible for some aspects of the survey.

Of the responding PDs, 14 (58%) reported including education on palliative care in their formal fellowship curriculum. There were no significant demographic differences between PDs who provide palliative education as part of their fellowship curriculum and those who do not (Supplementary Appendix SA3). Most PDs reported that their fellows provide palliative and end-of-life care to infants, and have access to a pediatric palliative care team.

Of the responding fellows, 30% reported receiving palliative care education as part of their training. The majority of the fellows were female, in their first or second year of fellowship, and their primary place of practice was in a level IV NICU. Most responding fellows reported providing palliative and end-of-life care to an infant. There were no significant demographic differences between fellows who reported receiving palliative care education and those who did not (Table 1).

**Table 1.** Demographic Information of Responding Fellows

	Formal training in palliative care		*p*
	No (*N* = 42), *n* (%)	Yes (*N* = 20), *n* (%)	
Gender			0.9999
Female	33 (79)	17 (85)	
Male	8 (19)	3 (15)	
No response	1 (2)	0 (0)	
Years training			0.1461
PGY 4	15 (36)	3 (15)	
PGY 5	18 (43)	9 (45)	
PGY 6	9 (21)	7 (35)	
PGY 7	0 (0)	1 (5)	
Level NICU			0.7140
III	6 (14)	4 (20)	
IV	36 (85)	16 (80)	
1. Have you ever provided palliative care to an infant—yes	36 (88)	20 (100)	0.1620
2. Have you ever provided end-of-life care to an infant—yes	40 (95)	20 (100)	0.9999
3. Do you have a pediatric palliative care team in your primary hospital?—yes	31 (74)	17 (95)	0.7174

NICU, neonatal intensive care unit; PGY, post-graduate year.

To determine how palliative education was provided, respondents were given a list of five formats and asked whether they were used in their training. The formats included lectures or seminars, clinical time with a palliative care provider, workshops, simulation or role-play, or self-study methods, including online modules or videos. All the PDs and 85% of fellows reported that lectures or seminars were used. Interestingly, 90% of the fellows reported the use of simulation or role-play, whereas only 57% of PDs reported the use of this format. Fellows were also more likely to report use of workshops (40%) and reliance on self-study (20%) as educational formats than PDs (21%, and 7%, respectively). Seventy-one percent of PDs reported spending time with a palliative care provider as part of the educational experience, compared with only 40% of the fellows (Supplementary Appendix SA4).

Fellows who received formal palliative care education, compared with those who did not, were more comfortable with several important aspects of palliative care (Table 2), including leading goals-of-care conversations (35% vs. 2%, *p* = 0.001), breaking bad news (40% vs. 14%, *p* = 0.048), discussing change in code status (35% vs. 10%, *p* = 0.029), grief and bereavement (25% vs. 5%, *p* = 0.031), and managing respiratory symptoms at the end of life (30% vs. 7%, *p* = 0.026).

**Table 2.** Fellows Who Self-Reported Being “Very Comfortable” with Key Areas of Palliative Care

	Formal training in palliative care		*p*
	No (*N* = 42)	Yes (*N* = 20)	
	*n* (%)	*n* (%)	
1. Leading goals-of-care conversations	1 (2)	7 (35)	0.001
2. Breaking bad news to parents/families	6 (14)	8 (40)	0.0478
3. Discussing changing the code status of a patient	4 (10)	7 (35)	0.0286
4. Discussing treatment withholding or withdrawal	4 (10)	4 (20)	0.4184
5. Transition to home hospice	0 (0)	1 (5)	0.3226
6. Grief and bereavement	2 (5)	5 (25)	0.0306
7. Providing antenatal counseling for extreme prematurity	13 (31)	10 (50)	0.1694
8. Identifying life-limiting conditions	6 (14)	3 (15)	0.9999
9. Providing counseling regarding the prognosis of life-limiting conditions	1 (2)	3 (15)	0.0945
10. Assessing for and managing pain at the end of life	3 (7)	5 (25)	0.0984
11. Assessing for and managing respiratory symptoms at the end of life	3 (7)	6 (30)	0.0255

## Discussion

Our data show that the majority (58%) of neonatology fellowship PDs reported providing formal palliative care education in some format. However, of the responding fellows, 70% reported that palliative care education was not part of their training. The cause of this disparity, whether actual or perceived, is unclear. There were some differences in the types of educational formats identified by fellows and PDs as used in their education. Fellows were more likely to report simulation, role-play, and workshops as educational formats than PDs, but less likely to report spending time with a palliative care provider. Previous studies have highlighted similar inconsistencies. A 2003 study of 62 medical schools found that although the majority of the faculty felt education in end-of-life care was very important for medical students (75%) and residents (93%), the students and residents perceived this very differently. Only 28% of medical students and 40% of residents believed that the attendings felt it was important. Despite this difference in perception, the majority of respondents felt that inclusion of a formal curriculum would improve palliative care education.^6^ Understanding this discrepancy is important, as most of the responding fellows (80%) reported being uncomfortable or only somewhat comfortable with all 11 key areas of palliative care; however, almost all responding fellows reported providing infants with palliative and end-of-life care.

The provision of excellent palliative care requires a broad range of skills that go beyond providing medications or procedural interventions. Although competency in pain and symptom management are important, what sets palliative care apart from other fields of medicine is the focus on high-level communication skills and psychosocial support. Bereaved parents report that insensitivity, lack of emotional support, and poor communication from care providers, and in particular from physicians, has a lasting impact on how they experience their grief, and on their long-term healing.^15,16^ Although a growing number of neonatologists recognize the role that palliative care can play in ensuring excellent end-of-life care, most have received little or no formal training.^10^ Importantly, trainees in our study who received formal education self-reported greater comfort in the high-level communication skills that are most closely tied to the palliative care specialty, specifically goals-of-care conversations, breaking bad news, and discussing infant code status changes. Skills that allow providers to compassionately support and guide families through these difficult times must be taught and developed in our fellows.

Fortunately, studies show that the provision of formal training can increase provider comfort level not only with end-of-life care, but also with general principles of palliative care^17,18^ that can be applied in other clinical situations. A variety of teaching methods, including didactics, small group discussion, role-play, and standardized patient experiences, have been employed with the goal of improving these communication skills in trainees.^11^ However, studies on the efficacy of simulation have rarely focused on pediatric fellows, or on the long-term impact of educational sessions.^19^ The most successful educational programs rely on a combination of methods, and the ability to practice communication skills regularly is essential for long-term retention.^9^ The principles of effective communication, specifically strategies for leading goals-of-care conversations and breaking bad news, are hallmarks of excellent palliative care, and are also integral to several skills that are fundamental to neonatology, especially antenatal counseling at the limit of viability. Despite this, only a minority of neonatal–perinatal training programs utilize simulation and role-modeling in teaching critical skills.^20^

This study aimed at determining whether palliative care education is provided to neonatal fellows as a formal part of their training, and at determining the impact that this education has on the fellows who receive it. Interestingly, although more than half of PDs reported that palliative care is included as a formal part of the curriculum, less than a third of responding fellows reported receiving palliative care education. This suggests a disconnect between what we think we are teaching our fellows, and what they are actually perceiving. Considering this disconnect may be important for how fellowship PDs approach palliative care education in their programs. Since not all fellows have similar learning styles, teaching may need to be tailored to best meet the fellow's needs. After palliative care education has been given, seeking fellow feedback may be essential to determine how the fellow perceived the teaching experience, what learning opportunities still exist, and what is their comfort level regarding various palliative care situations. Promoting a “recognized educational experience” through timely feedback may reinforce learning and modify perceptions regarding palliative care education. This view is supported by our survey, which demonstrated that fellows who recognize palliative care education do report a benefit from it, especially with regard to communication skills.

As with all studies reliant on survey responses, this study has several limitations. With a PD response rate of 26%, there is the possibility of bias toward programs that include palliative care education in their curriculum, or whose PDs have an interest in this topic. This may also bias the views of their fellows. The fellow response rate was better, at 33%; however, this was an imperfect measurement, and it represents only a small portion of neonatal fellows overall. Therefore, these data cannot be considered conclusive and may not be representative of neonatal programs and fellows as a whole. The discrepancy between how PDs and fellows reported exposure to palliative care education may simply be due to the inherent limitations of a survey study; however, it warrants further investigation. Finally, we developed and used a survey tool that was tested on a panel of University of Wisconsin-Madison neonatology faculty and fellows, but it was not validated. Our rationale for using our survey was that we were unaware of any current validated tools specific to neonatology trainees that addressed our areas of interest involving palliative care in the NICU setting. Future studies, ideally using a validated survey tool, will be important to verify our results.

## Conclusion

Most responding fellows (80%) reported being uncomfortable or only somewhat comfortable with all 11 key areas of palliative care. Fellows who reported receiving palliative care education were more confident in their abilities in many of these areas, suggesting that formal palliative care education can be effective at teaching fellows these vital skills. We advocate that all neonatology fellowship programs should have robust palliative care education curriculums to provide fellows the proper tools to navigate challenging conversations surrounding a dying baby. These learned skills can improve the fellow's comfort level with these difficult situations and will likely lead to better care for dying babies and their families. Future research should focus on determining the best ways to teach fellows palliative care skills that not only improve self-reported comfort levels, but also emphasize objective assessments of trainee ability.

## Supplementary Material

Supplemental data

Supplemental data

Supplemental data

Supplemental data

## Supplementary Material

Supplementary Appendix SA1

Supplementary Appendix SA2

Supplementary Appendix SA3

Supplementary Appendix SA4

## Abbreviations Used

ABP: American Board of Pediatrics
NICU: neonatal intensive care unit
PC: palliative care
PD: program director
PGY: post-graduate year
