# Supplementary material for: Gaps in Palliative Care Education among Neonatology Fellowship Trainees
Source: Palliat Med Rep. 2021 Jul 27;2(1):212–7. doi: 10.1089/pmr.2021.0011 (PMC8675219; doi:10.1089/pmr.2021.0011)
Supplement: Supplemental data [file Supp_AppS1.docx]

**Appendix A1:** Survey tool

1. The survey questions are slightly different depending on your role. Please choose one of the following options: *(this question is required)*
   1. Neonatology Fellow >> *go to fellow survey questions*
   2. Neonatology Fellowship Program Director >> *go to PD survey questions*

Questions for Neonatology Fellows:

1. Do you have a pediatric palliative care program in your hospital?
   1. Yes
   2. No
   3. Unsure
2. Have you ever provided palliative care to an infant?
   1. Yes
   2. No
3. Have you ever provided end-of-life care to an infant?
   1. Yes
   2. No
4. Do you receive education in palliative care?
   1. Yes
   2. No >> *skip to question 7*
5. Are any of the following formats currently used for palliative care education in your program? Yes/No
   1. Lectures or seminars
   2. Clinical time with palliative care provider
   3. Online modules, videos, or other self-study
   4. Workshops
   5. Simulation or role-play
6. On a scale from 1-5 with 1= very uncomfortable, 3= neutral, and 5= very comfortable, please state how comfortable you are with the following elements of palliative care:
   1. Leading goals of care conversations
   2. Breaking bad news to parents/families
   3. Discussing changing the code status of a patient
   4. Discussing treatment withholding or withdrawal
   5. Transition to home hospice
   6. Grief and bereavement
   7. Providing antenatal counseling for extreme prematurity
   8. Identifying life-limiting conditions
   9. Providing counseling regarding the prognosis of life-limiting conditions
   10. Assessing for and managing pain at the end-of-life
   11. Assessing for and managing respiratory symptoms at the end-of-life
7. On a scale from 1-5, with 1= Strongly Disagree, 3= neither agree nor disagree, 5= Strongly agree, how much do you agree with the following statements?
   1. Palliative care is the same as giving up
   2. Palliative care should only be used when an infant is dying and there are no other options
   3. Palliative care can be used in conjunction with curative therapies
   4. When a fetus is diagnosed with a life-limiting condition the option for palliative care at delivery should only be offered if pregnancy termination is declined or not an option
   5. When a fetus is diagnosed with a life-limiting condition the option for palliative care at delivery should be discussed concurrently with pregnancy termination
   6. Parents should be involved in the end of life decisions for their infants
   7. I have a particular interest in caring for infants at the end-of-life
   8. I am interested in further education in palliative care
8. Are there any palliative care topics that you would particularly like to have teaching in? – comment box
9. What is your gender?
   1. Female
   2. Male
10. What year of training are you?
    1. PGY-4
    2. PGY-5
    3. PGY-6
    4. PGY-7
11. What level nursery is your primary training site?
    1. I
    2. II
    3. III
    4. IV
12. In what state/region do you practice? (Drop down including Wash DC and Other options)
13. How many fellows are in your program? – comment box (optional)

Questions for Neonatology Program Directors:

1. Do you have a pediatric palliative care program in your primary hospital?
   1. Yes
   2. No
   3. Unsure
2. Do your fellows ever provide palliative care to infants?
   1. Yes
   2. No
3. Do your fellows ever provide end-of-life care to infants?
   1. Yes
   2. No
4. Is education on palliative care currently included in your fellow curriculum?
   1. Yes
   2. No >> *skip to question 8*
5. Are the following formats used to provide palliative care education? Yes/No
   1. Lecture(s) or seminars
   2. Clinical time with a palliative care team or provider
   3. Online modules, videos, or other self-study
   4. Workshops
   5. Simulation or role-play
6. Are following topics are covered in the training provided to your fellows? Yes/No
   1. Goals of care conversations
   2. Breaking bad news to parents/families
   3. Discussing changing the code status of a patient
   4. Discussing treatment withholding or withdrawal
   5. Transition to home hospice
   6. Grief and bereavement
   7. Providing antenatal counseling for extreme prematurity
   8. Identifying life-limiting conditions
   9. Providing counseling regarding the prognosis of life-limiting conditions
   10. Assessing for and managing pain at the end-of-life
   11. Assessing for and managing respiratory symptoms at the end of life
7. On a scale from 1-5, with 1= Strongly Disagree, 3= neither agree nor disagree, 5= Strongly agree, how much do you agree with the following statements?
   1. Palliative care is the same as giving up
   2. Palliative care should only be used when an infant is dying and there are no other options
   3. Palliative care can be used in conjunction with curative therapies
   4. Parents should be involved in the end-of-life decisions for their infants
   5. I have a particular interest in caring for infants at the end-of-life
   6. I am interested in further education in palliative care
8. Is there anything else you think we should know about the palliative care training for neonatal fellows at your program? – comment box
9. What is your gender?
   1. Female
   2. Male
10. How many years have you been out of fellowship?
    1. 0-5 years
    2. 6-15 years
    3. 16-25 years
    4. 26+ years
11. What level nursery do you primarily practice in?
    1. I
    2. II
    3. III
    4. IV
12. In what state/region do you practice? (Drop down including Wash DC and Other options)
13. How many fellows are in your program? – comment box
14. Did you send this survey to your fellows? Yes/No *(required questions)*
    1. If no, do you plan on sending this to your fellows? Yes/No
