# Supplementary material for: Gaps in Palliative Care Education among Neonatology Fellowship Trainees
Source: Palliat Med Rep. 2021 Jul 27;2(1):212–7. doi: 10.1089/pmr.2021.0011 (PMC8675219; doi:10.1089/pmr.2021.0011)
Supplement: Supplemental data [file Supp_AppS3.docx]

**Appendix A3:** Demographic information for responding program directors.

|  | **Palliative Care Education included in the fellowship curriculum** | | | |  |
| --- | --- | --- | --- | --- | --- |
|  | **No**  **(N=10)** | | **Yes**  **(N=14)** | |  |
|  | **N** | **%** | **N** | **%** | **p-value** |
| Gender  Female  Male | 6  4 | 60%  40% | 10  4 | 71%  29% | 0.6734 |
| Years in Practice  6-15 yrs  16-25 yrs  26+ yrs | 5  3  2 | 50%  30%  20% | 6  5  2 | 46%  38%  15% | 0.9999 |
| Level NICU  III  IV | 2  8 | 20%  80% | 2  11 | 15%  85% | 0.9999 |
| Do your fellows provide palliative care to infants? – Yes | 8 | 80% | 13 | 93% | 0.5504 |
| Do your fellows provide end-of-life care to infants? – Yes | 9 | 90% | 14 | 100% | 0.4167 |
| Do you have a pediatric palliative care team in your primary hospital? - Yes | 9 | 90% | 13 | 93% | 0.9999 |

NICU = neonatal intensive care unit
