# Supplementary material for: Gaps in Palliative Care Education among Neonatology Fellowship Trainees
Source: Palliat Med Rep. 2021 Jul 27;2(1):212–7. doi: 10.1089/pmr.2021.0011 (PMC8675219; doi:10.1089/pmr.2021.0011)

**Appendix A4:** Formats that fellows and PDs report are used to provide palliative care education during Neonatology training.


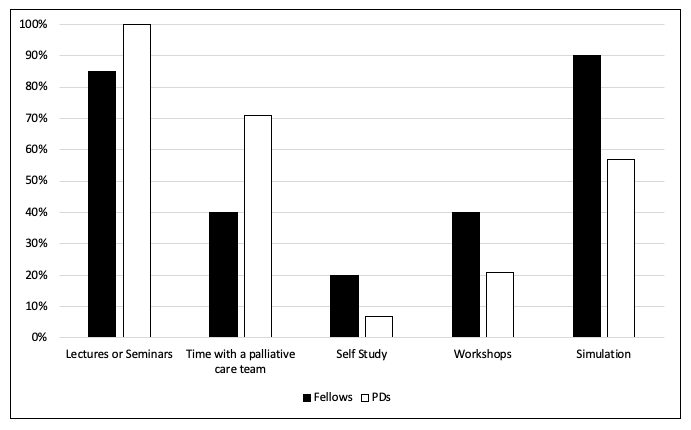

Supplement: Supplemental data [file Supp_AppS4.docx]
